# Supplementary material for: Long-term changes as oil palm plantation age simplify the structure of host-parasitoid food webs
Source: PLoS One. 2023 Oct 10;18(10):e0292607. doi: 10.1371/journal.pone.0292607 (PMC10564177; doi:10.1371/journal.pone.0292607)
Supplement: S1 Table — These matrices were used to calculate metrics for trophic interaction networks, which were analyzed using a bipartite ecological network approach (Dorman et al., 2009). Plot codes were derived from Table 1 in the text. (DOCX) [file pone.0292607.s001.docx]

**Plot 31**

| Species | Braconidae.005 | Aulosaphes.sp | Braconidae.137 | Elasmus.sp | Eulophinae.2 | Encyrtidae.3 | Eulophidae.09 | Eulophidae.10 | Ichneumonidae.08 | Ichneumonidae.30 |
| --- | --- | --- | --- | --- | --- | --- | --- | --- | --- | --- |
| Birthamula.chara | 0 | 0 | 0 | 0 | 0 | 0 | 0 | 0 | 0 | 0 |
| Clania.tertia | 0 | 0 | 1 | 1 | 0 | 0 | 0 | 0 | 1 | 1 |
| Darna.diducta | 0 | 0 | 0 | 0 | 0 | 0 | 0 | 0 | 0 | 0 |
| Darna.trima | 0 | 0 | 0 | 0 | 0 | 0 | 0 | 0 | 0 | 0 |
| Geometridae.1 | 0 | 0 | 0 | 0 | 0 | 0 | 0 | 0 | 0 | 0 |
| Geometridae.2 | 0 | 0 | 0 | 0 | 0 | 0 | 0 | 0 | 0 | 0 |
| Geometridae.3 | 0 | 0 | 0 | 0 | 0 | 0 | 0 | 0 | 0 | 0 |
| Geometridae.4 | 0 | 0 | 0 | 0 | 0 | 0 | 0 | 0 | 0 | 0 |
| Geometridae.5 | 0 | 0 | 0 | 0 | 0 | 0 | 0 | 0 | 0 | 0 |
| Lymantriidae.02 | 0 | 0 | 0 | 0 | 0 | 0 | 0 | 0 | 0 | 0 |
| Lymantriidae.03 | 0 | 0 | 0 | 0 | 0 | 0 | 0 | 0 | 0 | 0 |
| Lymantriidae.04 | 0 | 0 | 0 | 0 | 0 | 1 | 1 | 0 | 0 | 0 |
| Lymantriidae.05 | 0 | 0 | 0 | 0 | 0 | 0 | 0 | 0 | 0 | 0 |
| Lymantriidae.07 | 0 | 0 | 0 | 0 | 0 | 0 | 0 | 0 | 0 | 0 |
| Lymantriidae.08 | 0 | 0 | 0 | 0 | 0 | 0 | 0 | 0 | 0 | 0 |
| Lymantriidae.09 | 0 | 0 | 0 | 0 | 0 | 0 | 0 | 0 | 0 | 0 |
| Lymantriidae.10 | 0 | 0 | 0 | 0 | 0 | 0 | 0 | 0 | 0 | 0 |
| Lymantriidae.11 | 0 | 0 | 0 | 0 | 0 | 0 | 0 | 0 | 0 | 0 |
| Mahasena.corbetti | 0 | 6 | 0 | 0 | 0 | 0 | 0 | 0 | 0 | 0 |
| Metisa.plana | 0 | 3 | 0 | 9 | 2 | 0 | 0 | 0 | 1 | 1 |
| Pteroma.pendula | 1 | 0 | 1 | 0 | 0 | 0 | 0 | 10 | 0 | 0 |
| Setora.nitens | 0 | 0 | 0 | 0 | 0 | 0 | 0 | 0 | 0 | 0 |

**Plot 32**

| Species | Fornicia.sp | Braconidae.137 | Elasmus.sp | Eulophinae.2 | Ichneumonidae.08 | Ichneumonidae.30 |
| --- | --- | --- | --- | --- | --- | --- |
| Birthamula.chara | 0 | 0 | 0 | 0 | 0 | 0 |
| Birthosea.bisura | 0 | 0 | 0 | 0 | 0 | 0 |
| Clania.tertia | 0 | 0 | 5 | 1 | 1 | 0 |
| Darna.diducta | 0 | 0 | 0 | 0 | 0 | 0 |
| Darna.trima | 5 | 0 | 0 | 0 | 0 | 0 |
| Geometridae.1 | 0 | 0 | 0 | 0 | 0 | 0 |
| Geometridae.2 | 0 | 0 | 0 | 0 | 0 | 0 |
| Geometridae.3 | 0 | 0 | 0 | 0 | 0 | 0 |
| Geometridae.5 | 0 | 0 | 0 | 0 | 0 | 0 |
| Geometridae.6 | 0 | 0 | 0 | 0 | 0 | 0 |
| Lymantriidae.01 | 0 | 0 | 0 | 0 | 0 | 0 |
| Lymantriidae.02 | 0 | 0 | 0 | 0 | 0 | 0 |
| Lymantriidae.03 | 0 | 0 | 0 | 0 | 0 | 0 |
| Lymantriidae.04 | 0 | 0 | 0 | 0 | 0 | 0 |
| Lymantriidae.06 | 0 | 0 | 0 | 0 | 0 | 0 |
| Lymantriidae.08 | 0 | 0 | 0 | 0 | 0 | 0 |
| Lymantriidae.12 | 0 | 0 | 0 | 0 | 0 | 0 |
| Lymantriidae.13 | 0 | 0 | 0 | 0 | 0 | 0 |
| Lymantriidae.14 | 0 | 0 | 0 | 0 | 0 | 0 |
| Mahasena.corbetti | 0 | 1 | 6 | 0 | 0 | 0 |
| Metisa.plana | 0 | 1 | 4 | 0 | 0 | 1 |
| Pteroma.pendula | 0 | 1 | 0 | 0 | 0 | 0 |
| Setora.nitens | 0 | 0 | 0 | 0 | 0 | 0 |

**Plot 33**

| Species | Bethylidae.1 | Braconidae.005 | Fornicia.sp | Braconidae.137 | Eulophinae.2 |
| --- | --- | --- | --- | --- | --- |
| Clania.tertia | 0 | 1 | 0 | 0 | 0 |
| Darna.diducta | 1 | 0 | 0 | 0 | 0 |
| Darna.trima | 0 | 0 | 10 | 0 | 0 |
| Lymantriidae.01 | 0 | 0 | 0 | 0 | 0 |
| Lymantriidae.10 | 0 | 0 | 0 | 0 | 0 |
| Mahasena.corbetti | 0 | 0 | 0 | 0 | 0 |
| Metisa.plana | 0 | 0 | 0 | 2 | 1 |
| Pteroma.pendula | 0 | 0 | 0 | 2 | 0 |
| Setora.nitens | 0 | 0 | 0 | 0 | 0 |

**Plot 61**

| Species | Braconidae.032 | Aulosaphes.sp | Elasmus.sp | Ichneumonidae.45 |
| --- | --- | --- | --- | --- |
| Clania.tertia | 0 | 0 | 1 | 1 |
| Darna.trima | 0 | 0 | 0 | 0 |
| Geometridae.3 | 0 | 0 | 0 | 0 |
| Lymantriidae.03 | 0 | 0 | 0 | 0 |
| Lymantriidae.08 | 1 | 0 | 0 | 0 |
| Lymantriidae.10 | 0 | 0 | 0 | 0 |
| Lymantriidae.11 | 0 | 0 | 0 | 1 |
| Lymantriidae.12 | 0 | 0 | 0 | 0 |
| Lymantriidae.15 | 0 | 0 | 0 | 0 |
| Mahasena.corbetti | 0 | 11 | 0 | 0 |
| Metisa.plana | 0 | 5 | 0 | 2 |
| Pteroma.pendula | 0 | 0 | 0 | 0 |

**Plot 62**

| Species | Braconidae.032 | Fornicia.sp | Aulosaphes.sp | Eulophinae.2 | Eulophidae.08 | Ichneumonidae.45 |
| --- | --- | --- | --- | --- | --- | --- |
| Birthamula.chara | 0 | 0 | 0 | 0 | 0 | 0 |
| Clania.tertia | 0 | 0 | 0 | 0 | 0 | 0 |
| Darna.diducta | 0 | 5 | 0 | 0 | 0 | 0 |
| Lymantriidae.03 | 0 | 0 | 0 | 0 | 0 | 0 |
| Lymantriidae.08 | 1 | 0 | 0 | 0 | 0 | 0 |
| Lymantriidae.11 | 0 | 0 | 0 | 0 | 0 | 0 |
| Lymantriidae.12 | 0 | 0 | 0 | 0 | 5 | 0 |
| Lymantriidae.13 | 0 | 0 | 0 | 0 | 0 | 0 |
| Lymantriidae.15 | 0 | 0 | 0 | 0 | 0 | 0 |
| Mahasena.corbetti | 0 | 0 | 6 | 0 | 0 | 1 |
| Metisa.plana | 0 | 0 | 0 | 0 | 0 | 1 |
| Pteroma.pendula | 0 | 0 | 1 | 1 | 0 | 0 |
| Setora.nitens | 0 | 0 | 0 | 0 | 0 | 0 |

**Plot 63**

| Species | Aulosaphes.sp | Eulophinae.2 | Ichneumonidae.45 |
| --- | --- | --- | --- |
| Clania.tertia | 0 | 0 | 0 |
| Darna.diducta | 0 | 0 | 0 |
| Geometridae.2 | 0 | 0 | 0 |
| Lymantriidae.08 | 0 | 0 | 0 |
| Lymantriidae.12 | 0 | 0 | 0 |
| Mahasena.corbetti | 9 | 0 | 1 |
| Metisa.plana | 0 | 1 | 0 |
| Pteroma.pendula | 0 | 0 | 0 |
| Setora.nitens | 0 | 0 | 0 |

**Plot 101**

| Species | Eurytomidae.1 |
| --- | --- |
| Birthosea.bisura | 0 |
| Clania.tertia | 0 |
| Darna.diducta | 5 |
| Geometridae.2 | 0 |
| Geometridae.3 | 0 |
| Geometridae.5 | 0 |
| Geometridae.7 | 0 |
| Geometridae.8 | 0 |
| Lymantriidae.03 | 0 |
| Lymantriidae.10 | 0 |
| Lymantriidae.11 | 0 |
| Mahasena.corbetti | 0 |
| Metisa.plana | 0 |

**Plot 102**

| Species | Aulosaphes.sp |
| --- | --- |
| Clania.tertia | 0 |
| Darna.trima | 0 |
| Lymantriidae.11 | 0 |
| Mahasena.corbetti | 3 |
| Metisa.plana | 0 |
| Pteroma.pendula | 0 |

**Plot 103**

| Species | Fornicia.sp | Aulosaphes.sp | Elasmus.sp |
| --- | --- | --- | --- |
| Clania.tertia | 0 | 0 | 1 |
| Darna.diducta | 60 | 0 | 0 |
| Lymantriidae.03 | 0 | 0 | 0 |
| Lymantriidae.08 | 0 | 0 | 0 |
| Lymantriidae.11 | 0 | 0 | 0 |
| Mahasena.corbetti | 0 | 5 | 0 |
| Metisa.plana | 0 | 0 | 1 |
| Setora.nitens | 0 | 0 | 0 |
