# Supplementary material for: Long-term changes as oil palm plantation age simplify the structure of host-parasitoid food webs
Source: PLoS One. 2023 Oct 10;18(10):e0292607. doi: 10.1371/journal.pone.0292607 (PMC10564177; doi:10.1371/journal.pone.0292607)
Supplement: S2 Table — These matrices were used to obtain the metrics of trophic interaction networks, which were analyzed using a bipartite ecological network (Dorman et al., 2009). (DOCX) [file pone.0292607.s002.docx]

**Jambi1**

| Species | Eulophidae.sp3 | Ichneumonidae.sp5 | Ichneumonidae.sp6 |
| --- | --- | --- | --- |
| Darna.trima | 0 | 1 | 0 |
| Geometridae.sp1 | 10 | 0 | 1 |
| Hesperiidae.sp2 | 0 | 0 | 0 |
| Limantriidae.sp3 | 0 | 0 | 0 |

**Jambi2**

| Species | Braconidae.sp1 | Braconidae.sp2 | Eulophidae.sp1 | Eulophidae.sp2 | Eurytomidae.sp1 | Ichneumonidae.sp1 | Ichneumonidae.sp2 |
| --- | --- | --- | --- | --- | --- | --- | --- |
| Darna.trima | 0 | 0 | 0 | 0 | 0 | 0 | 0 |
| Geometridae.sp1 | 0 | 0 | 0 | 0 | 0 | 0 | 0 |
| Hesperiidae.sp1 | 0 | 0 | 0 | 0 | 0 | 0 | 0 |
| Limantriidae.sp1 | 0 | 0 | 0 | 0 | 0 | 1 | 1 |
| Limantriidae.sp3 | 0 | 0 | 244 | 0 | 0 | 0 | 0 |
| Nympalidae.sp1 | 0 | 0 | 0 | 0 | 0 | 0 | 0 |
| Parasa.lepida | 0 | 59 | 0 | 0 | 0 | 0 | 0 |
| Psychidae.sp1 | 0 | 0 | 0 | 0 | 0 | 0 | 0 |
| Setora.nitens | 4 | 0 | 0 | 43 | 1 | 0 | 0 |
| Setothosea.asigna | 0 | 0 | 0 | 0 | 0 | 0 | 0 |

**Jambi3**

| Species | Braconidae.sp1 | Braconidae.sp3 |
| --- | --- | --- |
| Darna.trima | 0 | 0 |
| Limantriidae.sp1 | 0 | 0 |
| Limantriidae.sp2 | 0 | 39 |
| Nympalidae.sp1 | 0 | 0 |
| Parasa.lepida | 0 | 0 |
| Setora.nitens | 1 | 0 |
| Setothosea.asigna | 0 | 0 |
