# Supplementary material for: Long-term changes as oil palm plantation age simplify the structure of host-parasitoid food webs
Source: PLoS One. 2023 Oct 10;18(10):e0292607. doi: 10.1371/journal.pone.0292607 (PMC10564177; doi:10.1371/journal.pone.0292607)
Supplement: S3 Table — Plot codes were based on Table 6 in the text. (DOCX) [file pone.0292607.s003.docx]

| **Family** | **Morphospecies** | **P121** | **P122** | **P141** | **P142** | **P161** | **P162** | **P171** | **P172** | **P181** | **P182** |
| --- | --- | --- | --- | --- | --- | --- | --- | --- | --- | --- | --- |
| **Bethylidae** | Bethylidae.01 | 1 |  |  |  |  |  |  |  |  |  |
|  | Bethylidae.02 |  | 1 |  |  |  |  |  |  |  |  |
|  | Bethylidae.03 |  |  |  | 1 |  |  |  |  |  |  |
| **Braconidae** | Braconidae.001 | 1 |  |  |  | 1 |  |  |  |  | 1 |
|  | Braconidae.002 |  |  |  |  |  |  |  |  |  | 1 |
|  | Braconidae.003 |  |  |  |  |  |  | 1 |  |  |  |
|  | Braconidae.004 | 1 |  |  |  | 2 |  |  |  | 1 |  |
|  | Braconidae.005 |  |  |  |  |  |  | 3 |  |  |  |
|  | Braconidae.006 |  |  |  | 2 |  |  |  |  |  |  |
|  | Braconidae.007 | 1 |  |  |  |  |  |  |  |  |  |
|  | Braconidae.008 |  |  |  |  |  | 1 |  |  |  |  |
|  | Braconidae.009 | 1 |  |  |  | 8 |  |  | 6 |  |  |
|  | Braconidae.010 |  |  |  | 1 |  |  |  |  |  |  |
|  | Braconidae.011 | 2 |  |  |  |  | 1 |  |  |  |  |
|  | Braconidae.012 |  |  |  |  | 1 |  |  |  |  |  |
|  | Braconidae.013 | 3 | 5 | 3 | 5 | 1 | 3 | 3 | 9 | 7 | 4 |
|  | Braconidae.014 |  | 1 |  |  |  |  |  |  |  |  |
|  | Braconidae.015 |  | 1 |  | 1 |  |  |  |  |  |  |
|  | Braconidae.016 |  |  |  |  |  |  | 1 |  |  |  |
|  | Braconidae.017 | 1 |  |  |  |  | 1 |  | 1 |  |  |
|  | Braconidae.018 |  |  |  |  |  |  | 2 |  |  |  |
|  | Braconidae.019 | 1 |  | 3 | 1 | 1 | 2 | 2 |  | 1 | 1 |
|  | Braconidae.020 |  |  | 3 |  |  |  |  |  |  |  |
|  | Braconidae.021 | 1 |  |  |  |  |  |  |  |  |  |
|  | Braconidae.022 | 1 |  |  | 1 |  | 1 |  | 1 |  | 2 |
|  | Braconidae.023 |  | 1 |  |  |  |  | 2 | 2 | 1 |  |
|  | Braconidae.024 | 1 | 4 | 4 | 4 | 1 |  | 1 |  | 3 | 4 |
|  | Braconidae.025 | 7 | 8 | 2 | 2 | 1 | 1 | 1 | 2 | 6 | 2 |
|  | Braconidae.026 |  |  |  |  |  |  |  | 1 |  |  |
|  | Braconidae.027 |  | 3 |  | 1 |  |  |  |  |  |  |
|  | Braconidae.028 |  |  | 1 |  | 2 |  |  |  | 1 |  |
|  | Braconidae.029 | 1 | 2 |  | 2 |  | 1 | 1 | 2 | 7 |  |
|  | Braconidae.030 |  | 2 |  |  |  |  |  |  |  |  |
|  | Braconidae.031 |  |  | 1 |  | 1 |  |  |  |  |  |
|  | Braconidae.032 | 6 | 3 | 1 | 7 | 1 | 2 | 2 | 1 | 9 | 10 |
|  | Braconidae.033 |  | 1 |  | 1 |  |  |  |  |  |  |
|  | Braconidae.034 |  |  |  |  |  |  |  | 1 |  |  |
|  | Braconidae.035 |  | 1 |  |  | 1 |  | 1 |  | 1 |  |
|  | Braconidae.036 | 1 |  |  |  |  |  |  |  |  |  |
|  | Braconidae.037 | 17 | 7 | 1 | 7 |  |  | 1 | 1 | 2 |  |
|  | Braconidae.038 |  | 1 | 1 | 3 |  |  |  |  |  |  |
|  | Braconidae.039 |  | 1 |  |  |  |  |  |  |  |  |
|  | Braconidae.040 | 1 |  |  |  |  |  |  |  |  |  |
|  | Braconidae.041 | 1 |  |  |  |  |  |  |  | 1 |  |
|  | Braconidae.042 |  | 2 | 2 | 2 | 3 | 1 | 1 | 2 | 3 |  |
|  | Braconidae.043 | 1 |  |  |  |  |  |  | 1 | 1 |  |
|  | Braconidae.044 |  |  |  |  |  | 1 |  |  |  |  |
|  | Braconidae.045 |  |  |  |  | 1 |  |  |  |  |  |
|  | Fornicia.sp | 14 | 8 | 12 | 5 | 7 | 9 | 10 | 7 | 26 | 6 |
|  | Braconidae.047 |  |  |  |  |  |  |  |  |  |  |
|  | Braconidae.048 |  |  | 1 | 2 |  |  |  |  | 1 |  |
|  | Braconidae.049 | 8 | 28 | 3 | 19 | 6 | 3 | 16 | 10 | 6 | 14 |
|  | Braconidae.050 |  |  |  |  |  |  |  | 1 |  |  |
|  | Aulosaphes.sp |  |  |  | 1 |  |  |  |  |  |  |
|  | Braconidae.052 |  | 1 |  |  |  |  |  |  |  |  |
|  | Braconidae.053 |  |  |  | 1 |  |  |  |  |  |  |
|  | Braconidae.054 |  | 1 |  |  |  |  |  |  |  |  |
|  | Braconidae.055 |  |  |  | 1 | 1 | 2 |  |  |  |  |
|  | Braconidae.056 | 2 |  |  |  |  |  |  |  |  |  |
|  | Braconidae.057 |  |  |  | 3 |  |  |  |  |  |  |
|  | Braconidae.058 |  |  |  |  |  |  |  | 1 |  |  |
|  | Braconidae.059 | 1 | 1 | 1 | 2 | 2 | 3 | 1 | 9 | 3 | 1 |
|  | Braconidae.060 |  |  |  | 1 |  |  |  |  |  |  |
|  | Braconidae.061 |  |  |  |  |  |  |  | 1 |  |  |
|  | Braconidae.062 | 1 |  |  |  |  |  |  |  |  |  |
|  | Braconidae.063 |  |  |  |  |  |  |  |  |  | 1 |
|  | Braconidae.064 |  | 1 |  |  |  |  |  |  |  |  |
|  | Braconidae.065 |  | 1 |  | 2 | 2 |  | 1 | 1 |  |  |
|  | Braconidae.066 |  |  |  |  | 1 |  |  |  |  |  |
|  | Braconidae.067 |  |  |  |  |  |  |  | 1 |  |  |
|  | Braconidae.068 |  |  |  | 1 | 1 |  |  |  | 1 | 1 |
|  | Braconidae.069 |  |  |  | 1 |  |  |  |  |  |  |
|  | Braconidae.070 |  |  |  |  |  |  |  | 1 | 1 |  |
|  | Braconidae.071 |  |  |  |  | 1 |  |  |  |  |  |
|  | Braconidae.072 |  | 1 |  | 1 |  |  | 1 |  |  |  |
|  | Braconidae.073 |  |  | 1 |  |  |  |  |  |  |  |
|  | Braconidae.074 |  |  |  | 3 |  |  |  |  |  | 1 |
|  | Braconidae.075 |  | 2 |  | 1 |  |  |  |  |  |  |
|  | Braconidae.076 |  |  |  | 1 |  |  |  | 1 |  |  |
|  | Braconidae.077 |  |  |  | 1 |  |  |  |  |  |  |
|  | Braconidae.078 | 3 |  |  |  |  |  |  |  | 1 |  |
|  | Braconidae.079 | 7 |  | 2 | 1 | 3 | 11 | 2 | 2 | 5 |  |
|  | Braconidae.080 | 2 | 1 |  | 1 | 1 | 1 | 1 | 1 | 1 |  |
|  | Braconidae.081 |  |  |  |  | 1 |  |  |  |  |  |
|  | Braconidae.082 |  |  |  |  | 1 |  | 1 |  |  |  |
|  | Braconidae.083 |  |  | 1 |  |  |  |  |  |  |  |
|  | Braconidae.084 | 5 | 3 |  | 2 | 4 |  |  | 5 | 1 |  |
|  | Braconidae.085 |  |  |  |  | 3 |  |  |  |  |  |
|  | Braconidae.086 |  |  |  | 1 |  |  |  |  |  |  |
|  | Braconidae.087 |  | 2 | 1 | 1 | 2 | 2 | 2 | 2 | 2 | 1 |
|  | Braconidae.088 | 1 | 6 | 1 |  | 1 |  |  |  | 1 | 1 |
|  | Braconidae.089 |  |  |  | 1 |  |  |  |  | 1 |  |
|  | Braconidae.090 |  |  |  | 2 |  |  |  |  | 3 |  |
|  | Braconidae.091 |  |  |  | 2 |  |  |  |  |  |  |
|  | Braconidae.092 |  |  |  |  | 1 |  |  |  |  |  |
|  | Braconidae.093 | 1 |  |  |  |  | 1 |  |  |  |  |
|  | Braconidae.094 |  |  |  | 1 |  |  |  |  |  |  |
|  | Braconidae.095 | 1 | 3 | 3 | 11 | 1 | 1 | 4 | 3 | 1 | 4 |
|  | Braconidae.096 |  | 1 | 1 | 1 |  |  |  |  |  | 1 |
|  | Braconidae.097 | 1 |  |  |  | 1 |  |  |  |  |  |
|  | Braconidae.098 |  |  | 4 |  |  |  |  |  |  |  |
|  | Braconidae.099 |  |  | 3 | 1 | 4 |  | 1 |  |  | 1 |
|  | Braconidae.100 |  |  |  |  | 1 |  |  |  |  |  |
|  | Braconidae.101 |  |  |  | 1 |  |  |  |  |  |  |
|  | Braconidae.102 |  |  |  |  |  |  |  |  |  |  |
|  | Braconidae.103 | 4 | 3 | 5 | 14 | 16 | 2 | 5 | 1 | 16 |  |
|  | Braconidae.104 | 1 |  |  |  |  |  |  |  |  |  |
|  | Braconidae.105 |  |  |  |  |  |  |  |  | 1 |  |
|  | Braconidae.106 | 1 |  |  |  | 1 |  |  |  |  |  |
|  | Braconidae.107 |  |  |  |  |  |  |  |  |  |  |
|  | Braconidae.108 |  |  |  |  | 1 | 1 |  |  |  | 6 |
|  | Braconidae.109 | 1 |  |  |  |  |  |  |  |  |  |
|  | Braconidae.110 | 5 | 1 |  | 10 | 7 | 2 | 1 | 1 | 4 | 1 |
|  | Braconidae.111 | 1 |  |  | 2 | 1 |  |  |  | 3 |  |
|  | Braconidae.112 |  |  |  |  | 2 | 1 |  |  |  |  |
|  | Braconidae.113 |  |  | 1 |  |  | 3 |  |  |  | 5 |
|  | Braconidae.114 |  |  |  |  |  | 1 |  |  |  |  |
|  | Braconidae.115 |  |  |  |  |  |  |  |  | 1 |  |
|  | Braconidae.116 |  |  |  |  |  |  |  |  | 1 |  |
|  | Braconidae.117 |  |  |  |  |  |  | 1 | 1 | 1 |  |
|  | Braconidae.118 |  |  |  |  |  |  |  | 1 |  |  |
|  | Braconidae.119 |  |  |  |  |  |  | 1 |  |  |  |
|  | Braconidae.120 | 1 |  |  | 1 |  | 1 |  |  | 3 |  |
|  | Braconidae.121 |  |  |  |  |  |  |  |  |  |  |
|  | Braconidae.122 |  |  | 1 |  |  | 1 |  |  | 2 |  |
|  | Braconidae.123 |  |  |  |  |  |  |  |  |  |  |
|  | Braconidae.124 | 9 | 13 | 10 | 5 | 22 | 15 | 39 | 31 | 25 | 30 |
|  | Braconidae.125 | 7 | 2 | 1 | 5 | 1 |  | 3 | 2 | 4 |  |
|  | Braconidae.126 | 2 | 2 | 2 | 2 | 1 | 1 | 2 | 3 |  | 1 |
|  | Braconidae.127 | 1 |  |  |  |  |  |  |  |  |  |
|  | Braconidae.128 |  |  |  |  |  |  |  |  | 1 |  |
|  | Braconidae.129 | 1 |  |  | 2 |  |  |  |  | 2 |  |
|  | Braconidae.130 |  | 5 |  | 2 | 1 | 1 |  | 2 | 4 | 3 |
|  | Braconidae.131 |  | 1 |  |  |  |  |  |  |  |  |
|  | Braconidae.132 | 2 | 6 | 9 |  | 2 | 1 | 1 |  | 2 | 2 |
|  | Braconidae.133 |  |  | 1 |  | 1 |  |  |  |  | 1 |
|  | Braconidae.134 | 1 | 1 | 1 |  |  |  |  | 1 |  | 2 |
|  | Braconidae.135 |  |  |  |  |  |  | 1 |  | 1 |  |
|  | Braconidae.136 |  |  |  |  |  |  |  |  |  | 1 |
| **Chalcididae** | Chalcididae.01 | 3 | 1 |  |  | 6 | 4 | 8 | 1 | 2 | 8 |
| **Diapriidae** | Diapriidae.01 | 1 | 2 | 1 | 3 | 1 |  |  | 2 | 1 |  |
|  | Diapriidae.02 |  |  |  |  | 1 |  |  |  |  |  |
|  | Diapriidae.03 |  |  |  |  |  |  |  |  | 1 |  |
|  | Diapriidae.04 |  |  |  |  |  |  |  |  |  |  |
|  | Diapriidae.05 |  |  |  |  |  |  |  |  |  |  |
|  | Diapriidae.06 | 5 |  |  | 1 |  |  |  |  | 2 |  |
|  | Diapriidae.07 |  |  |  |  |  |  |  |  | 1 |  |
|  | Diapriidae.08 |  |  |  |  |  |  | 1 |  |  |  |
|  | Diapriidae.09 | 1 |  |  |  |  |  |  |  |  |  |
|  | Diapriidae.10 |  | 1 |  |  |  |  |  |  |  |  |
|  | Diapriidae.11 | 1 |  |  |  |  |  |  |  |  |  |
|  | Diapriidae.12 | 1 |  |  |  |  |  |  |  |  |  |
|  | Diapriidae.13 |  |  |  |  |  |  |  |  | 1 |  |
| **Encyrtidae** | Encyrtidae.01 |  | 1 |  |  |  |  |  |  |  |  |
|  | Encyrtidae.02 | 2 |  |  |  |  |  |  |  |  |  |
| **Eucharitidae** | Eucharitidae.01 |  | 1 |  |  |  |  |  |  |  |  |
|  | Eucharitidae.02 |  |  |  |  |  |  |  | 1 |  |  |
| **Eucoilidae** | Eucoilidae.01 | 1 |  |  |  |  |  |  | 1 | 1 |  |
| **Eulophidae** | Eulophidae.01 |  |  |  |  |  | 1 |  |  |  |  |
|  | Eulophidae.02 |  |  |  | 1 |  |  |  |  |  |  |
|  | Eulophidae.03 |  |  |  |  |  |  |  | 1 |  |  |
|  | Eulophidae.04 |  |  |  |  | 1 |  |  | 2 | 3 |  |
|  | Eulophidae.05 |  | 3 |  | 1 |  |  |  |  |  |  |
|  | Eulophidae.06 |  |  |  |  | 1 |  |  |  |  |  |
|  | Eulophidae.07 | 1 |  |  |  |  |  |  |  |  |  |
| **Eupelmidae** | Eupelmidae.01 | 6 | 2 | 6 | 3 | 20 | 1 | 5 | 8 | 14 | 2 |
|  | Eupelmidae.02 |  |  |  |  |  |  |  |  |  | 4 |
| **Evaniidae** | Evaniidae.01 |  |  |  | 7 |  | 1 |  | 3 | 2 |  |
|  | Evaniidae.02 |  |  |  | 2 |  |  |  |  |  |  |
|  | Evaniidae.03 |  |  |  |  |  |  | 1 |  |  |  |
| **Ichneumonidae** | Ichneumonidae.01 |  |  |  |  | 4 |  | 1 |  | 1 | 1 |
|  | Ichneumonidae.02 |  | 2 |  |  |  |  | 3 |  | 1 |  |
|  | Ichneumonidae.03 |  |  |  |  |  | 3 |  |  |  |  |
|  | Ichneumonidae.04 |  |  |  |  | 1 |  |  |  |  |  |
|  | Ichneumonidae.05 |  |  |  |  |  |  | 1 |  |  | 1 |
|  | Ichneumonidae.06 | 1 |  |  |  | 1 | 1 |  | 1 | 1 |  |
|  | Ichneumonidae.07 |  |  |  |  |  | 1 |  |  |  |  |
|  | Ichneumonidae.08 | 2 | 8 | 7 | 4 | 6 | 2 | 12 | 5 | 5 | 3 |
|  | Ichneumonidae.09 | 5 | 10 | 17 | 7 | 9 | 5 | 24 | 18 | 5 | 13 |
|  | Ichneumonidae.10 |  |  |  |  |  |  |  | 1 | 1 | 2 |
|  | Ichneumonidae.11 | 1 | 2 | 3 | 2 | 3 | 3 | 8 | 2 | 7 | 1 |
|  | Ichneumonidae.12 |  | 1 |  |  |  |  | 1 |  |  |  |
|  | Ichneumonidae.13 |  |  |  |  |  |  |  | 2 |  |  |
|  | Ichneumonidae.14 | 1 |  |  |  |  |  |  |  |  |  |
|  | Ichneumonidae.15 | 3 |  | 2 | 4 |  |  | 1 | 2 | 1 | 3 |
|  | Ichneumonidae.16 |  |  |  | 2 |  |  |  |  | 1 |  |
|  | Ichneumonidae.17 |  | 5 | 1 | 1 | 8 | 1 | 1 |  |  | 1 |
|  | Ichneumonidae.18 |  |  |  | 1 |  |  |  |  |  |  |
|  | Ichneumonidae.19 |  | 1 |  |  |  |  | 1 |  |  |  |
|  | Ichneumonidae.20 |  |  |  |  |  |  |  | 2 | 1 | 1 |
|  | Ichneumonidae.21 | 1 |  |  |  |  |  |  |  | 1 |  |
|  | Ichneumonidae.22 |  |  |  |  |  |  |  | 1 |  |  |
|  | Ichneumonidae.23 |  |  |  |  | 2 |  |  |  |  |  |
|  | Ichneumonidae.24 | 1 | 1 |  |  | 4 | 1 |  |  | 1 | 3 |
|  | Ichneumonidae.25 | 1 |  |  |  |  |  |  |  |  |  |
|  | Ichneumonidae.26 | 2 | 14 | 5 | 5 | 28 | 6 | 12 | 21 | 4 | 9 |
|  | Ichneumonidae.27 | 6 | 9 | 2 | 1 | 44 | 11 | 20 | 37 | 22 | 30 |
|  | Ichneumonidae.28 | 2 | 4 |  | 9 | 3 |  | 3 | 12 |  | 1 |
|  | Ichneumonidae.29 |  |  |  |  | 1 |  |  | 1 | 1 |  |
|  | Ichneumonidae.30 | 3 | 2 |  |  |  |  | 1 | 1 |  |  |
|  | Ichneumonidae.31 |  |  |  |  |  |  |  | 1 |  |  |
|  | Ichneumonidae.32 |  |  |  |  | 1 | 1 |  |  |  |  |
|  | Ichneumonidae.33 |  |  |  | 1 | 2 |  | 2 |  |  | 2 |
|  | Ichneumonidae.34 |  |  |  |  | 2 |  |  |  |  |  |
|  | Ichneumonidae.35 | 7 | 4 | 2 | 1 |  | 4 | 3 | 3 | 3 | 2 |
|  | Ichneumonidae.36 | 6 |  | 1 | 1 | 8 | 1 | 1 | 2 | 2 | 5 |
|  | Ichneumonidae.37 |  |  |  | 2 | 1 |  |  |  |  |  |
|  | Ichneumonidae.38 |  |  |  |  | 2 |  |  |  |  |  |
|  | Ichneumonidae.39 |  |  |  |  |  |  |  |  |  | 1 |
|  | Ichneumonidae.40 |  |  |  |  | 1 |  |  |  |  |  |
|  | Ichneumonidae.41 |  |  |  |  |  |  |  | 1 |  |  |
|  | Ichneumonidae.42 |  |  |  |  |  |  | 1 |  |  |  |
|  | Ichneumonidae.43 |  |  |  |  | 1 |  | 1 |  |  |  |
|  | Ichneumonidae.44 |  |  |  | 1 | 1 |  | 1 |  |  |  |
| **Megaspilidae** | Megailidae.01 |  | 1 |  |  |  |  |  |  |  |  |
|  | Megailidae.02 |  |  |  |  |  |  | 1 |  |  |  |
| **Pteromalidae** | Pteromalidae.01 |  |  |  |  |  |  |  |  |  | 1 |
|  | Pteromalidae.02 |  |  |  |  | 1 | 9 |  |  |  | 1 |
| **Scelionidae** | Scelionidae.01 |  |  |  |  |  |  |  |  | 1 |  |
|  | Scelionidae.02 |  |  |  | 1 |  |  |  |  |  |  |
|  | Scelionidae.03 |  |  |  | 2 |  |  |  |  | 1 |  |
|  | Scelionidae.04 |  |  |  | 1 |  |  |  |  |  |  |
|  | Scelionidae.05 |  |  |  | 2 |  |  |  |  |  | 1 |
|  | Scelionidae.06 |  |  | 3 | 4 |  | 1 | 1 | 2 | 2 |  |
|  | Scelionidae.07 |  |  |  |  |  |  |  |  |  |  |
|  | Scelionidae.08 | 2 | 4 | 2 | 5 | 1 |  | 2 | 5 | 1 |  |
|  | Scelionidae.09 | 1 | 1 |  | 1 |  |  |  |  |  |  |
|  | Scelionidae.10 |  |  |  |  | 1 | 1 |  |  |  |  |
|  | Scelionidae.11 |  |  |  |  |  |  |  |  | 1 |  |
|  | Scelionidae.12 |  |  |  |  | 1 |  |  |  | 1 |  |
|  | Scelionidae.13 |  |  |  |  |  |  |  |  |  | 1 |
| **Tetracampidae** | Tetracampidae.01 |  |  |  | 1 |  |  |  |  |  |  |
|  | Tetracampidae.02 |  |  | 1 |  | 1 | 1 | 2 |  | 1 | 1 |
|  | Tetracampidae.03 |  |  |  |  |  | 1 |  |  |  |  |
|  | Tetracampidae.04 |  |  |  |  |  |  |  |  | 1 |  |
|  | Tetracampidae.05 |  | 5 |  | 1 |  |  |  |  |  |  |
|  | Tetracampidae.06 |  |  |  | 1 |  |  |  |  |  |  |
