# Supplementary material for: Long-term changes as oil palm plantation age simplify the structure of host-parasitoid food webs
Source: PLoS One. 2023 Oct 10;18(10):e0292607. doi: 10.1371/journal.pone.0292607 (PMC10564177; doi:10.1371/journal.pone.0292607)
Supplement: Attachment — Submitted filename: Response to reviewers.docx [file pone.0292607.s004.docx]

Dear Reviewers,

We would like to resubmit our manuscript ID PONE-D-22-26542 entitled “Long-term tree growth of oil palm simplifies the structure of host-parasitoid food webs" for publication in PLOS ONE.

We thank you for the constructive comments and we have revised the manuscript accordingly. In this version, we revised the title to “Long-term change of oil palm plantation simplifies the structure of host-parasitoid food webs”.

This document contains your comments and our response in italics. Where we thought recommended changes were not appropriate, we give the reason in the response.

We hope you will find our manuscript suitable for publication in PLOS ONE.

Sincerely,

Akhmad Rizali

---------------------------------

Reviewer #1:

This interesting paper compares characteristics of lepidopteran and parasitoid species richness and food webs across different ages of oil palm, with sites in Central Borneo and Jambi Province, Sumatra. The study includes data from direct collection of larvae and raising of parasitoids from earlier ages of oil palm, and collections of larvae and parasitoids by insecticide fogging in later ages.

The work represents an enormous collection of data, providing a comprehensive overview of the effects of oil palm age on lepidopteran larvae, parasitoids, and their interactions. The key findings are that number of species of larvae, parasitoids and linkage density all decline with oil palm age in earlier years (from direct collection data), but this is not apparent in later years (from fogging data). This finding has important implications for understanding the ecology of oil palm systems and informing pest management approaches.

I felt that the study set-up was robust and the reporting fair, but I did find it quite hard to follow exactly how the study was carried out in places, particularly related to the analyses, and that the results could be discussed in more detail. I therefore recommend the changes below, which I hope are helpful. My overarching suggestions are:

1) I don’t think using the terms “tree growth” or “development” are clear, as this suggests it is just tree size that is changing – I would suggest rewording to “plantation age” throughout, to encompass all the other environmental differences that occur as plantations mature.

*We preferred to change the title with “Long-term change of oil palm plantation simplifies the structure of host-parasitoid food webs”. But in the whole manuscript, we have replaced the terms “tree growth” with “tree age” or “plantation age”.*

2) Please add more details to the methods about the two study sites and why these were chosen for comparison – this isn’t really clear at the moment.

*We have added the explanation in the methods “We selected Central Kalimantan and Jambi due to two regions have similar topography and climate that mainly lowland area and with tropical climates.”*

3) Please give specific details of what factors were included in each analysis and whether assumptions of tests were assessed and met. I couldn’t really follow what was included in the different tests from the methods.

*The main objective of this research is to study the effect of tree age and environment condition (understorey vegetations) on host-parasitoid food webs. We have added more detail about the factors in the method section*

4) I wasn’t sure how Shannon was being calculated in this case (e.g. just for parasitoids?) Please add details to the methods.

*To study the host-parasitoid food webs, we quantified using bipartite ecological networks that was developed by Dormann et al (2009, The Open Ecology Journal. 2:7-24). The bipartite analysis provided quantitative metrics from the food web, include of species number of LL (host), species number of HL (parasitoid), linkage density and Shannon diversity. Based on Dormann et al (2009), linkage density was calculated based on marginal totals-weighted diversity of interactions per species (quantitative) which is computed as the average of vulnerability and generality (Bersier et al. 2002). While Shannon diversity is Shannon's diversity of interactions (i.e., network entries).*

*We have added this explanation in the method section.*

5) I would like to know more about the effectiveness of raising larvae – e.g. what was the level of survival to adulthood or emergence of parasitoids across different species etc. and percentage of parasitism? Could differing survival across species impact food-web results?

*We have added in Table 2 and Table 4 to show the list of lepidopteran larvae and their parasitoids as well as the percentage of parasitism.*

6) I think the paper would also be improved by including some sort of compositional analysis, to quantify how larval and parasitoid composition changed across ages. Abundance of pests and parasitoids can be important, as well as diversity, so I also suggest including analyses on total abundances.

*We have added a table to show species richness and abundance of lepidopteran larvae and their parasitoids. We prefer not to analysis the species composition due to it has already show in the food-webs.*

7) I didn’t find the discussion structure very easy to follow and I would recommend more discussion about the reasons for differences observed (e.g. what specific factors might be driving differences across ages?). I also suggest adding a specific section comparing the differing results between the hand collection and fogging studies.

*This study used two different methods i.e., hand-collection method and the fogging method. The hand-collection method was carried out at 9 plots with each plot the observations were conducted in 100 oil palm trees with the purpose: (1) to confirm that the parasitoid wasps are associated with their hosts (lepidopteran pests) and (2) to ensure that samples can be obtained sufficient because each oil palm trees are not always found lepidoptera larvae. In addition, due to this method is only possible for oil palms under 10 years old, we also conducted fogging method to get the information about parasitoid wasps and lepidopteran larvae from oil palm trees above 10 years old. This method was carried out on different plots/locations from hand-collection method i.e., in 10 plots with each plot selected 6 oil palm trees as sample units. However, the host-parasitoid food webs can only be analysed based on the data from hand-collection method.*

I include specific points below:

Abstract:

The term “long-term tree development” isn’t very clear. Suggest replacing with “oil palm age” It isn’t clear what the set-up is from the abstract and what ages were collected from Jambi – please give more details here.

*We have replaced the term “long-term tree development” with “long-term change of oil palm plantation” and we have also added the tree age of oil palm in Jambi*

“Results of the GLM analysis showed that the abundance of Fornicia sp was not affected by the tree age of oil palm but had a positive effect on the abundance of lepidopteran larvae.” Presumably, it is the number of larvae affecting the number of wasps rather than the other way round – please rewrite to reflect this.

*We have reanalysed the data and rewritten the sentence*

Introduction:

60-61 – I don’t think it is clear what “harmonious and balanced interactions” are – suggest replacing with more specific wording.

*We preferred to use “balance interaction” and delete “harmonious”*

65- I am not sure “relies on” is the right term here – suggest rewording to say that species and their interactions support ecosystem services.

*We have reworded with “The importance of conserving these interactions and associated processes, as well as the component species that associated with species interactions, such as pollination and biological control, supports ecosystem services"*

71- Suggest rewording to “compare across ages”, rather than “growth of oil palm trees”, as many other factors are changing as well as height that could influence these interactions

*We rephrased the sentence to “This paper investigates how the long-term change of oil palm plantation, specifically the tree age, effects host-parasitoid food webs”*

73 – “more robust description” is this compared to descriptive methods? Please clarify.

*No, this is not compared. We replaced to “more robust quantification”*

83 – “imbalance” is a bit difficult to quantify – suggest rewording to something more specific such as “lower numbers of predators”, or “non-target effects on natural enemies”.

*We have replaced to “non-target effects on natural enemies”*

86-87 – It would be useful to include more information here about results from studies comparing differences in communities across different ages of oil palm.

*We have added the information about the effect of oil palm tree age on insect communities*

93 – Suggest adding more justification here about why different regions included and chosen.

*We have added a sentence as justification*

Methods:

97 – In this section, please add a description of the habitat conditions in both regions for context (e.g. climate, levels of natural vegetation, oil palm age structure across the landscape etc.)

*We have added the description of climate*

103 -106 - Suggest moving mention of collection methods to later, after detailing the plot set-up. I think the term “visual observation” is a bit misleading, as it implies you just observed rather than collecting, perhaps use “hand-collection” instead?

*We have moved the collection methods in the next subheading and change the term “visual observation” with “hand-collection”*

108 – “randomly selected as sampling units” - suggest reword to “randomly selected within these plots as sampling units”

*We have revised the sentence*

109-112 – It would be useful to add a justification here of why a comparison across areas was carried out?

*We have added the explanation and justification in previous paragraph*

121 – “within a hundred trees” - I’m not totally clear what was sampled here – were all 100 trees in each plot sampled for 15 minutes? Please clarify.

*We have revised the sentence by replace with “...for all hundred trees” and explain the sampling time in the next sentence*

121 -123 – Suggest rewording to “We searched for lepidopteran larvae for a total of 15 minutes per tree, looking for larvae for 5 minutes in the top, middle, and bottom section of each canopy.” Presumably, you only searched the oil palm fronds themselves (ie not the epiphytes or anything else?)

*Yes, we only searched the oil palm canopy without epiphytes. We have rephrased the sentence*

130 – “feeding larvae.” – is this whether they fed between observations? Please clarify.

*We decided to delete this word*

140 – Was the sheet placed right next to the tree (ie would it also collect insects from the

trunk/epiphytes etc?)

*Yes, it was. Because it was difficult to avoid the kill insects from the trunk/epiphytes*

147 – “diversity and cover of vegetation” please add more details – was it percentage cover by eye, number of all understory species (including grasses) etc.?

*Yes, it was. We have added the description in the next sentence*

161 –Please clarify how Shannon diversity was calculated – was this done separately for parasitoids and larvae?

*To study the host-parasitoid food webs, we quantified using bipartite ecological networks that was developed by Dormann et al (2009, The Open Ecology Journal. 2:7-24). The bipartite analysis provided quantitative metrics from the food web, include of species number of LL (host), species number of HL (parasitoid), linkage density and Shannon diversity. Based on Dormann et al (2009), linkage density was calculated based on marginal totals-weighted diversity of interactions per species (quantitative) which is computed as the average of vulnerability and generality (Bersier et al. 2002). While Shannon diversity is Shannon's diversity of interactions (i.e., network entries).*

*We have added this explanation in the method section.*

161-162 – Were data from all 100 trees in each plot pooled for all time periods, prior to analyses? Please give details.

*Due to, the data per time period is limited. We pooled the data in each plot for all time periods prior to analysis*

166 – Please state what the specific dependent variables were. Presumably only Age 3 plantations were included for Central Borneo?

*We reanalysed using ANOVA and add the explanation about responses and explanatory variables which were used.*

168-170 – Please clarify what dependent and independent variables were included in the models? E.g. was age included as well as environmental factors in single models? What aspects of parasitoids (abundance/diversity etc?) were included? Please state in full here. From looking at the results, I think this included number of lepidopteran species, number of parasitoid species, and aspects of foodweb structure. I think it would be good to test effects on total abundance. Please clarify that diagnostic tests were carried out to test assumptions of the tests.

*We have added the responses and explanatory variables as well as the explanation about model assumption.*

Results:

179 -181 – This is really methods – please move to that section.

*We have moved to the methods section*

177-179 –Please also add summary stats on how many parasitoids raised, level of survival etc. It would be useful to also add a brief summary of the most common species.

*We have added the summary of parasitoids emerged and the parasitization level of each parasitoid*

184-185 – Please state the results more completely in the text. From looking at the table, only tree age comes out as being a significant predictor and it would be helpful to state this.

*We have added more description about the analysis results of GLM*

189 – “found in different tree ages” I’m not sure what you mean here – are these species found across all oil palm ages? Please clarify.

*Yes, these species were found across all oil palm ages. We have revised the sentences*

Figure 2 and associated text – It would be useful to know the number of larvae that weren’t parasitized, as well as survival of larva. If some species died more commonly, how likely is this to have affected your results?

*Based on the bipartite analysis (Dormann et al 2009), the data that were used for analysis were individual number of parasitoids emerged from each host (lepidopteran pest). We have added Table 2 to showed the detail of parasitisation level of each parasitoid.*

Figure 3 – It would be helpful to add a community analysis (e.g. nMDS) to quantify differences in communities of larvae and wasps across ages.

*We prefer to focus on bipartite analysis as a tool to quantify the ecological networks that had been used another researcher such as Tylianakis et al 2007, Nature 445:202-205. With the metric can show the structure of host-parasitoid food webs.*

208-209 – As above – it would be helpful to quantify how much this finding might be influenced by larval survival etc?

*We have added Table 4 to show the parasitization level of each parasitoid*

209 – 211 – Please give full results in text (including what doesn’t differ). Where a difference is present, please state the direction of difference.

*We have added more description in the text*

219-220 – Were Fornicia spp. the only parasitoids found in these ages? Please clarify.

*We have added a short description of parasitoid diversity from fogging method*

220-222 – Please move to methods.

*We have moved to method section*

Discussion

247-250 – I think it would be useful to discuss specifically what is it about the older palms that might lead to these differences?

*We have added a discussion sentence about the consequence of oil palm tree growth*

254-256 – But only in the case of the fogging results. This result could reflect differing abilities to detect differences between techniques. Suggest adding a discussion point related to this. I think this lack of effect for older palms could also relate to mature palms all being rather similar environmentally once the canopy has closed, compared to differences in the environment between years in younger palms. Again, suggest adding a discussion point related to this.

*We have added a discussion related to this*

261-271 – Why do you think that understory vegetation is not affecting parasitoids in this case? Please discuss.

*We have added a discussion*

282-283 – Although not directly tested in this study. As above, suggest carrying out multivariate tests to assess differences in composition.

*In this research, we are only focus on host-parasitoid food webs. And related to the species composition, we have shown in Table 2 and Table 4*

285-292 – Do differences in landscape structure etc. between the two sites vary and in the direction you would predict from your results? Please add details.

*We have added a description about the two regions in the methodology. And in this research, we did not analyse the landscape structure and only predict based on previous research.*

I hope that these comments help to develop this important paper.

*Thank you very much for the inputs and comments*

With all best wishes

Edgar Turner, Insect Ecology Group, Department of Zoology, University of Cambridge, UK.

---------------------------------

---------------------------------

Reviewer #2:

The manuscript brings relevant information about how host-parasitoid food webs change with the increasing age of plants in oil palm, which can help in the ecological management of pests.

The manuscript is well written, with clear and objective language. However, some information needs to be presented in the manuscript. I consider two main problems to be solved:

1 - The host-parasitoid interactions need to be reviewed and substantiated, as there are mistaken interactions.

*In this research, we only used the data of hand-collection method to develop host-parasitoid interaction. We have made sure that the parasitoid wasps emerged from the collected hosts (lepidopteran larvae)*

2 - Both the number of species and the abundance of lepidopterans and parasitoids needs to be considered in the statistical models to make the results clearer.

*We have revised the Table 1 and added more detail about abundance and species richness of hosts and parasitoids. Due to we only focused on the structure of food webs, we only used the metric of food webs analysis based on bipartite (we have described in the method section). In this case, the response variables are LL: species number of lepidopteran larvae in the network, HH: species number of parasitoids in the network, LD: linkage density, H’: Shannon diversity*

MATERIALS AND METHODS

L120 Were the instars of the caterpillars considered in the collection or were the caterpillars collected indiscriminately? Make it clear.

*All instars of lepidopteran larvae were collected. We have added more description to make it clearer*

L128 In the laboratory, were the insects raised in a controlled environment? Make it clear whether the photoperiod, humidity, and temperature conditions were controlled or natural.

*No, lepidopteran larvae were not reared in a controlled environment. We have added more description*

L140 About the nebulization parasitoid collection method: how to guarantee that the dead parasitoids collected on the white canvas were associated with lepidopterans? The parasitoids could be visiting the tree.

*With the fogging method, we could not make sure that the parasitoids were associated with lepidopteran larvae. Therefore, we only focus on species Fornicia sp (as case study) that has been known (based on hand-collection method) attack lepidopteran larvae and always found in all fogging plots (tree age range from 12 to 18 years old).*

L152 Lepidoptera and parasitoid species appear in tables in appendices S1 and S2 as morphospecies. It is necessary to mention whether all species were separated into morphospecies based on morphological characteristics.

*We have added a description about the identification process*

L160 It should be made clear how the linkage density was calculated, as well as the Shannon diversity.

*To study the host-parasitoid food webs, we quantified using bipartite ecological networks that was developed by Dormann et al (2009, The Open Ecology Journal. 2:7-24). The bipartite analysis provided quantitative metrics from the food web, include of species number of LL (host), species number of HL (parasitoid), linkage density and Shannon diversity. Based on Dormann et al (2009), linkage density was calculated based on marginal totals-weighted diversity of interactions per species (quantitative) which is computed as the average of vulnerability and generality (Bersier et al. 2002). While Shannon diversity is Shannon's diversity of interactions (i.e., network entries).*

*We have added this explanation in the method section.*

L169 Mention the packages used to build the models.

*We did not use the package for GLM analysis. We only used the package of bipartite to analyse the metric of host parasitoid food webs and we have described in the method section.*

RESULTS

I have several questions about the collected parasitoids and their interactions:

Diapriidae.1. They are known to be parasitoids of larvae and pupae of several Diptera families, Coleoptera larvae such as Staphylinidae and Psephenidae families, and ant larvae. They are also recognized as hyperparasitoids through Dryinidae and Tachinidae. They are not associated with lepidopteran hosts (Please see Masner, 2006 Familia Diapriidae - in Hanson and Gauld, 2006 Hymenoptera de la Región Neotropical). Therefore, the parasitoid obtained certainly did not emerge from the lepidopteran *Metisa plana*, and could have emerged from a host different from the caterpillars collected. It could have been accidentally collected or be hyperparasitoid via Tachinidae, for example. In this case, it is necessary to exclude the species from the analyses.

*Thank you for the correction. We have excluded this morphospecies*

Scelionidae.14: The parasitoids of this family are known to be idiobiont endoparasitoids of insect and spider eggs. The authors obtained 40 parasitoids that emerged from Lepidoptera.1. However, the methodology of this research was based on the collection of lepidopteran larvae from plants. How could the authors explain the presence of 40 egg parasitoids?

*We decided to excluded this morphospecies too*

Chrysidae.sp1. They are known to be parasitoids of Hymenoptera (symphytes and nest builders) and Phasmatodea (Kimsey, 2006 Familia Chrysididae - in Hanson and Gauld, 2006 Hymenoptera de la Región Neotropical). Therefore, the parasitoids obtained certainly did not emerge from the lepidopteran *Parasa lepida*. In this case, as well as in the case of Diapriidae.1, it is necessary to exclude it from the analyses.

*Thank you for the correction. We have excluded this morphospecies*

According to Noyes, 2019 (Universal Chalcidoidea Database) the Elasmidae family is currently considered as a tribe within Eulophinae (Eulophidae). Make corrections to tables and text.

*We have replaced Elasmidae to Eulophinae*

As shown in the previous questions, host-parasitoid interactions are not scientifically supported. I understand that establishing these interactions with most parasitoids at the family taxonomic level is something very difficult. However, misinterpreted interactions compromise results in food web studies.

I suggest that the authors prepare a scientifically based table for host-parasitoid interactions and place it in the Supporting Information.

*We have added the tables in the Supporting Information as well as added Table 2 and Table 4 to show the list of lepidopteran pests and their parasitoids*

In the tables in appendices S1 and S2, all parasitoids are identified at the family level. However,

throughout the text, the authors pointed out some genera of the parasitoids obtained. It is necessary to add the identified parasitoid genera.

*If possible, some morphospecies were identified to genera level. We have added the description in the method section as well as replaced the family name to genera name in the appendices*

L 184. The authors mention that the increasing age of oil palm trees caused a decrease in the number of species of lepidopteran larvae and parasitoids. Subsequently, the authors showed that the abundance of lepidopteran larvae had a positive effect on the abundance of parasitoids. This suggests that the effect of increasing the age of trees on the decrease in parasitoid species may be an indirect effect mediated by the reduction in the abundance and number of Lepidoptera species. I believe that the authors should consider the abundance and number of lepidopteran and parasitoid species both in the models in which plant ages were tested and in the models in which parasitoid abundance was tested.

*We have reanalysed the data to provide the similar model with the data of hand-collection methods*

L 200 Fig 2 It is necessary to explain the size of the bars and links.

*We have added more description to explain the interaction graph*

---------------------------------
