# Supplementary material for: Long-term changes as oil palm plantation age simplify the structure of host-parasitoid food webs
Source: PLoS One. 2023 Oct 10;18(10):e0292607. doi: 10.1371/journal.pone.0292607 (PMC10564177; doi:10.1371/journal.pone.0292607)
Supplement: Attachment — Submitted filename: Response to reviewers.docx [file pone.0292607.s005.docx]

Dear Reviewer,

We would like to resubmit our manuscript ID PONE-D-22-26542R1 entitled “Long-term tree growth of oil palm simplifies the structure of host-parasitoid food webs" for publication in PLOS ONE.

We thank you for the constructive comments and we have revised the manuscript accordingly. In this version, we revised the title to “Long-term changes as oil palm plantation age simplify the structure of host-parasitoid food webs”.

This document contains your comments and our response in italics. Where we thought recommended changes were not appropriate, we give the reason in the response.

We hope you will find our manuscript suitable for publication in PLOS ONE.

Sincerely,

Akhmad Rizali

---------------------------------

Reviewer #1:

This interesting and data-rich manuscript is much clearer and most of my previous comments have been well-addressed. However, I still think that some sections would benefit from more clarifying, and I have some additional suggestions that I hope will help to improve the manuscript.

1) It was really helpful to see the abundance of Lepidoptera and parasitoids included in the table for section one (the hand collection), but it would be really useful to analyse changes in abundance through a GLM framework as well – it feels like this is missing from the manuscript at the moment. This is important, especially as the total number of larvae may have a bigger effect on herbivory than number of larval species, and I recommend this analysis is included. I can see why it may not make sense to do this for parasitoids, as these were raised from the larvae, and therefore may not reflect genuine abundance in the field (it would still be useful to state why this isn’t analysed in the text of the methods).

*We have added the GLM result of lepidopteran larvae abundance in the Table 3*

2) Related to this, I would also recommend analysing number of larval species (if available?) in section two of the study (the fogging). I think this would be really helpful, as it would mean that the same factors, as far as possible, are being analysed against habitat characteristics in both sections. If larvae weren’t identified to species because of limited time or other constraints, then this could be stated in the methods.

*Thank you for the recommendation. Unfortunately, we did not identify the specimens of lepidopteran larvae to species level due to very difficult and need long time to be conducted.*

3) I don’t think is it clear enough why Fornicia was focussed on (rather than all parasitoids) in the second section. I also wasn’t completely sure whether it was Fornicia abundance or number of species (I think the later) that was analysed? I think this should be clarified in the methods and results. In this case, I think abundance could also be analysed, as the fogging will be picking up a reasonable estimation of the number of parasitoids present. If only Fornicia were identified for time/pragmatic reasons, then I recommend this is stated in the methods.

*We have reanalysed the data with included the abundance of all parasitoid wasps. And in the methodology, we also have added the reason why Fornicia was also included in the analysis.*

4) I am still unclear how successful raising the larvae was and how many died (but not as a result of parasitization)? I know from experience that this can be quite high (often due to fungal infections and things) and it would be useful to have this information in the paper, if available. Related to this, was % parasitism calculated as “number of larvae with parasitoids emerging/number of larvae that successfully emerged to adulthood or had parasitoids emerged” or “number of larvae with parasitoids emerging/total number of larvae brought back” I guess the former, but I think this should be explicitly stated, as it could make quite a big difference to the percentage.

*We have added the data of (i) number of collected larvae and (ii) number of % parasitized larvae in Table 2. In this research, we did not observe other factors that affecting the died of lepidopteran larvae during the rearing in the laboratory, except the died because of parasitized by parasitoids.*

5) Finally, I recommend that the negative relationship between understory cover and lepidopteran larval species richness is discussed in the discussion. It would be good to add this as a short paragraph, as it has interesting management implications. Related to this, it would be useful to discuss briefly why number of understory plant species did not affect anything.

*We reanalysed the data and we found understorey vegetation are not significant both in hand-collecting method (P=0.090) and fogging method (P=0.085)*

Other than that, my specific comments are:

Title – I still don’t feel this is quite clear enough at first read. Suggest tweak wording to “Long-term changes as oil palm plantations age simplify the structure of host-parasitoid food webs”?

*We have revised the title*

Abstract.

35 – “Shannon diversity” I think non-specialists will think of simple diversity indices for each group when reading this, as I did. To clarify, I suggest you reword to “Interaction diversity”, clarifying that this was calculated through Shannon's in the methods. I think this will be clearer for a wider readership.

*We agree to reword “Shannon diversity” to “interaction diversity”*

39-41 – “Different geographical regions showed different host parasitoid food webs structure, especially species number of lepidopteran larvae and Shannon diversity.” please tweak the wording to say in which direction number of species and Shannon diversity differed.

*We have revised the sentence*

41-43 – “Based on fogging method, Fornicia sp (Hymenoptera: Braconidae) were recorded in a different tree age range from 12 to 18 years.” This isn’t quite clear – suggest rewording to “For example, Fornicia sp (Hymenoptera: Braconidae) were recorded in all ages of oil palm sampled.” As I think them being found across a range of ages is the key point here?

*We have reworded*

64 – I’m not completely sure what “balanced interactions” are – can you be more specific in the terminology here?

*We have revised to “stability of interaction networks”*

Introduction

82-83 – As above, suggest specifying how Shannon’s used in this case.

*We have reworded to interaction diversity*

Methods

119-120 – I think that “and without observation of parasitoid wasp diversity and lepidopteran larvae abundance.” Makes it a bit confusing what was measured. Suggest stating what was measured instead, perhaps “In this area, we selected three plots with trees aged three years and recorded host-parasitoid food webs, using the same methods applied in Central Kalimantan.”

*We have revised the sentence*

130 – “Borneo” – you use Kalimantan, Central Kalimantan, Central Borneo, and Borneo at different points in the manuscript. Suggest simplifying to “Kalimantan” throughout for clarity (especially for those not familiar with the region).

*We agree to change Borneo to Kalimantan*

133 – “with natural environmental conditions” –reword to “at room temperature” for clarity, as other conditions very different from the field.

*We have reworded*

151-152 – As the sheet was next to the tree, suggest adding “As the sheet was next to the tree, collections would likely include insects collected from both the oil palm canopy and from epiphytes growing on the trunk”, as a small caveat.

*We have added the sentence*

161-162 – Suggest adding a line to clarify what data was generated from this species identification. Perhaps: “From this we calculated number of understory species per quadrat” or something like that?

*We have revised the sentence*

175 – As earlier, suggest you give more details here of exactly how linkage density and Shannon diversity are calculated in this case, for non-experts.

*We have added the description*

185 – Suggest rewording “With assumed” to “Assuming”

*We have reworded*

189 – By “diversity” do you mean number of species of understory plants? Please tweak wording to clarify.

*We have reworded*

192 “from the data of fogging method, we studied the effect of explanatory variables on Fornicia sp and lepidopteran abundance.” – As these variables were calculated from the raw data, rather than from the outputs of the bipartite ecological network, I think this should be moved to the end of the “Insect identification” section, above where you talk about the bipartite ecological network. Perhaps something like “From the fogging data, we calculated Lepidoptera larval abundance and the number of Fornicia sp per plot” Was this carried out per plot or per tree? I wasn’t sure whether it was Fornicia sp number of species or abundance calculated? Please clarify. In the section “Observation of Parasitoid Wasp Diversity and Lepidopteran Larvae Abundance”, I think you need to say why data on Fornicia only used, rather than all parasitoids. Presumably, this is because this was the commonest group and found in reasonable abundance, so you are using this as an indicator of parasitoids? Please state in the methods to make sure it is clear.

*We have moved the sentence in the suggested section. And in this revised version, we included the abundance of all parasitoid wasps in the analysis, beside the abundance of Fornicia sp.*

195 – “metric of networks” - for clarity, suggest you specifically state the metrics compared here again.

*We have added the detail of network metrics*

Results

206 – 208 – suggest adding overall parasitisation rates here as well.

*We have added in the previous sentence*

211 “lepidopteran larvae was also affected by cover of understorey vegetation” please say in which direction this relationship was (negative)

*We have revised the sentence*

228 – Table 2 legend – include mention of parasitisation level in the legend, and briefly say how this was calculated (% of individual larvae with parasitoids emerging per species?). I am not sure whether this is calculated for all larvae or just larvae that survived to pupation/had parasitoid emergence – I think this should be clarified. Please also include mention of total number of plots represented in the legend, to allow the table to be interpreted without referring back to the text

*We have added the detail in the legend*

Table 2 – Please write abbreviations like “No. par” in full to avoid confusion

*We have written in full in the table*

232 – Table 3 legend – as above, please give details of number of plots in the legend.

*We have added the number of plots*

236-243 – As above, give details of number of plots represented in the figure legends

*We have added the number of plots*

249-250 – Rather than just significantly different, state which is higher/lower in terms of Lepidoptera species number and Shannon diversity in Jambi versus Kalimantan for clarity.

*We have revised the sentence*

253 -254 – Table 4 legend – Please give details of the number of plots in the legend, as above for other tables. Write abbreviations like “No. par” in full to avoid confusion.

*We have revised the legend and written in full in the table*

257 – Table 5 legend – please give details of number of plots in the legend.

*We have added the number of plots*

270-272 – As above – please say in which direction this marginal effect is (ie it is a negative association).

*We have revised the sentence*

262-277 – Section “Effect of host abundance, tree age, and understorey vegetation on parasitoid abundance”. As above, I am not totally sure whether it is number of Fornicia species or abundance– I think the former. Please clarify.

*We have revised the sentence and included all parasitoid wasps in the analysis*

278 – Table 7 legend – as above, please give N values in legend.

*We have added the value*

Discussion

299 -309 – Suggest adding a line or two to the end of this section, stating that the different results between the two sections of the study could be due to different methods and metrics used, with direct collection and raising of parasitoids likely to be more sensitive to the effects of oil palm age.

*We have added a sentence*

310-311 – “understorey vegetation, also did not affect the structure of host-parasitoid food webs in oil palm plantations.” Although number of larvae species did decrease with more cover? I think this should be added in here and discussed briefly, as it has interesting management implications.

*We have discussed in the paragraph*

332-342 – It would be useful to add some brief details of what the two study regions were like in the methods – ie did they differ in amount of forest cover etc, as you suggest could be driving results here?

*We have added the different condition of oil palm plantations between Central Kalimantan and Jambi*

Conclusion

355-368 – I think the findings also suggest that it might be best to replant oil palm in smaller blocks, creating a more diverse age-structure in oil palms?

*We did not analyse about the effect of block size and the diverse age-structure on the host-parasitoid food web. Therefore, we do not suggest in the conclusion.*

I hope that these additional comments help with this interesting and important paper.

*Thank you very much for your inputs and comments*

Edgar Turner, Insect Ecology Group, Department of Zoology, University of Cambridge.

---------------------------------
