# Supplementary material for: Long-term changes as oil palm plantation age simplify the structure of host-parasitoid food webs
Source: PLoS One. 2023 Oct 10;18(10):e0292607. doi: 10.1371/journal.pone.0292607 (PMC10564177; doi:10.1371/journal.pone.0292607)
Supplement: Attachment — Submitted filename: Response to reviewers.docx [file pone.0292607.s006.docx]

Dear Reviewer,

We would like to resubmit our manuscript ID PONE-D-22-26542R2 entitled “Long‐term changes as oil palm plantation age simplify the structure of host‐parasitoid food webs" for publication in PLOS ONE.

We thank you for the constructive comments and we have revised the manuscript accordingly. We also have did proofreading to improve the quality of our manuscript.

This document contains your comments and our response in italics. Where we thought recommended changes were not appropriate, we give the reason in the response.

We hope you will find our manuscript suitable for publication in PLOS ONE.

Sincerely,

Akhmad Rizali

---------------------------------

Reviewer #1:

I’m sorry to be slow doing this review. The authors have addressed my comments

thoroughly and I’m happy for this paper to be accepted. It contributes valuable and detailed

information about the ecological impacts of oil palm aging, and has important implications for pest management in these systems. If of help, I have some minor suggestions:

Methods – 147 – “similar heights” it would be good to give heights in text.

*We did not measure exactly the heights of oil palm trees on each plot. This statement emphasizes that similar height of oil palm trees within a plot and may different with the height of oil palm trees with other plots*

Table 1 – I think HL in the table should be HH?

*We have replaced HH to HL in the title of Table 1*

Table 3 – please clarify what LL and HH means in the figure legend for this too, or rephrase to

“Abundance of lepidopteran larvae” “Number of species of lepidopteran larvae” etc. for clarity.

*We have added the description in the title of Table 3 and also added the abbreviation in the title of Figure 1*
